# Tocilizumab-induced drug reaction with eosinophilia and systemic symptoms (DRESS) in a patient with rheumatoid arthritis

**DOI:** 10.1093/rap/rky029

**Published:** 2018-09-17

**Authors:** Rose I Massolino, Pravin Hissaria, Anita Lee, Susanna M Proudman

**Affiliations:** 1 Discipline of Medicine, University of Adelaide, Adelaide, South Australia, Australia; 2 Department of Clinical Immunology, Royal Adelaide Hospital, Adelaide, South Australia, Australia; 3 Rheumatology Department, Royal Adelaide Hospital, Adelaide, South Australia, Australia

**Key message**

- Consider drug reaction with eosinophilia and systemic symptoms as a side-effect of tocilizumab.

Sir, we report a case of probable drug reaction with eosinophilia and systemic symptoms (DRESS) after exposure to tocilizumab, a biologic DMARD typically used for RA.

Our patient was a non-smoker with a history of asthma, carpal tunnel syndrome, non-alcoholic fatty liver disease and seropositive RA.

She was treated with a combination of conventional synthetic DMARDs, initially MTX and HCQ; doses were modified or other DMARDs added according to a treat-to-target strategy aiming for low disease activity (DAS28-ESR) score of <3.2. Clinical remission was achieved for only 2 years with LEF before peripheral neuropathy developed and it was ceased. HCQ was eventually stopped owing to the development of blurred vision. Ciclosporin was then trialled but resulted in alopecia and was ceased. Adalimumab was trialled, but there was no response. She then tried etanercept, but this resulted in severe fatigue and joint swelling. AZA was trialled next, but it caused nausea and severe diarrhoea. She remained on MTX throughout this time.

**Fig. 1:**
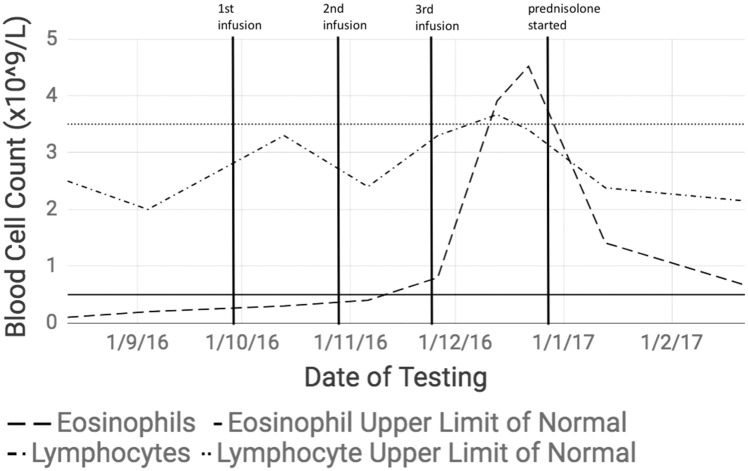
Eosinophil and lymphocyte counts during and after tocilizumab treatment, compared with upper limits of normal Eosinophil and lymphocyte counts are depicted compared with their upper limits. Dates of the first, second and third infusions of tocilizumab are marked. Elevations of eosinophil and lymphocyte counts are demonstrated, with a clear temporal relationship to commencement of tocilizumab treatment. The lymphocyte count progresses from a moderately high range and peaks slightly above the upper limit of normal, whereas the eosinophilia peaks well above the upper limit of normal. Both counts peak after the third infusion and normalize after cessation of the drug and commencement of prenisolone.

By August 2016, 12 years after her diagnosis, her DAS28 score was 4.53 (moderate disease activity), and she commenced tocilizumab at the protocol dose of 8 mg/kg/month. She was not experiencing any adverse drug effects at the time of initiation. She was still using MTX at the time, but no other new drugs. After two infusions, she was in DAS28 remission (2.34). After the first infusion, a rash developed that resolved with an antihistamine. On day 18 after the third infusion, a new eosinophilia of 3.91 × 10^9^/l (0.02–0.5 × 10^9^/l) and mild lymphocytosis of 3.67 × 10^9^/l (1.5–3.5 × 10^9^/l) were noted (Fig. 1). A follow-up appointment was booked to review these abnormal results 26 days after the third infusion. Here, she reported that she had become unwell since the third infusion, with anorexia, nausea, diarrhoea, extensive pruritus and epigastric pain, and weight loss of 5 kg. She experienced an exacerbation of asthma. She was afebrile. There were no abnormal lymphocytes reported on blood film, and her ESR and CRP remained normal. There was a transient and insignificant rise in alanine aminotransferase.

At 27 days, her eosinophil count had increased to a peak of 4.57 × 10^9^/l. She was due for her fourth infusion on day 28, but this was cancelled. Biochemistry, serum protein electrophoresis, tryptase and faecal screens for viruses, bacteria and parasites were negative. Serum antibodies for CMV, EBV and HHV-6 virus were negative. She started prednisolone 50 mg orally mane on day 35, weaning by 12.5 mg every 5 days. Her clinical picture and blood counts normalized after 8 weeks.

This is the first reported case of probable tocilizumab-induced DRESS. This rare and potentially life-threatening reaction is typically seen with antiepileptic agents and allopurinol [1]. Hallmark symptoms include fever, eosinophilia, rash and internal organ involvement. The mortality rate is 1.7% from end-organ failure, myocarditis or haemophagocytosis [1]. The pathophysiology of DRESS is complex, involving a type IVb hypersensitivity reaction. The drugs involved in these reactions may directly interact with T-cell receptors to stimulate an anti-drug immune response [2]. Furthermore, evidence of viral reactivation of HHV-6, CMV and EBV is common [3, 4], suggesting that the drug causes a viral reactivation, leading to an anti-viral immune response. In this patient, despite the lack of evidence of viral reactivation, the combination of eosinophilia, lymphocytosis and gastrointestinal involvement suggests DRESS; the lymphocytosis makes DRESS more likely than hypereosinophilia.

Tocilizumab is a monoclonal antibody to the IL-6 receptor (IL-6R) that blocks the pro-inflammatory effect of IL-6 by binding to these receptors. Tocilizumab has not been associated with reactivation of CMV, EBV or HHV-6, but has been associated with HBV reactivation [5]. The large number of drugs implicated in this syndrome suggests that it is likely to be mediated by the host response to the drug, rather than a specific effect of the drug itself. A similar case of eosinophilia and abdominal pain with eosinophilic infiltrate and microabcesses in response to tocilizumab has been reported as drug-induced hypereosinophilia [6]. It now seems pertinent to question whether this, too, is a case of DRESS.

There is an association between tocilizumab and DRESS, although the aetiology remains unknown. Given that DRESS is a serious condition, patients should be educated about the symptoms, and health-care professionals should be aware of this reaction in those commencing tocilizumab.

*Funding*: No specific funding was received from any bodies in the public, commercial or not-for-profit sectors to carry out the work described in this manuscript.

*Disclosure statement*: The authors have declared no conflicts of interest.
